# Introducing the new culture section of *BJPsych Bulletin*

**DOI:** 10.1192/bjb.2020.128

**Published:** 2021-02

**Authors:** David Foreman

**Affiliations:** Institute of Psychiatry, Psychology & Neuroscience, King's College London, UK

**Keywords:** Psychiatry, culture, mental health, patient services

## Abstract

This editorial launches the new culture section in the journal. Without any unchallengeable definition of ‘culture’, potential contributors may consider submissions under four headings: the arts and humanities relating to practice; regulatory culture; becoming a cultured practitioner; and psychiatry's cultural context. A new article type, ‘Cultural reflections’, has been created, and submissions may reflect any appropriate methodology, including those from the arts. Peer review (from methodologies outside psychiatry if appropriate) will assure quality. Our objectives are to establish *BJPsych Bulletin* as the ‘journal of record’ for cultural studies relevant to psychiatric service delivery and demonstrate equivalent quality between them and scientific studies.

The ‘mission statement’ of *BJPsych Bulletin* appears at the top of its Instructions for authors. It reads, ‘*BJPsych Bulletin* prioritises research, opinion and informed reflection on the state of psychiatry, management of psychiatric services, and education and training in psychiatry’.

We try to provide what psychiatrists need to practise well. With our daily professional lives governed by scientific evidence and policy delivery, the utility of audits, guidance reviews, clinical recommendations and service-related research is obvious. However, we believe that psychiatrists also need excellent cultural understanding and culturally informed practice to deliver what our patients need from us. We have therefore created a new Cultural Section, with an associated article type, ‘Cultural reflections’, to allow submission of articles that do not fit the currently available Journal frameworks.

## What do we mean by culture?

Like baldness or serious professional misconduct, culture is something we have little trouble recognising but great difficulty defining. Jahoda observed that the definitions of culture commonly used in psychological science are mutually incompatible, not amenable to empirical testing and suggests that we define it by usage rather than semantically.^1^ As editors of the journal, if someone wishes to submit something they consider ‘cultural’, we recommend that they think in terms of four headings: the arts and humanities concerning practice; regulatory culture; the cultured practitioner; and the cultural context within which psychiatry operates.

### The arts and humanities concerning psychiatric practice

The arts and humanities are what we usually think of when ‘culture’ is discussed. However, while a vibrant psychiatric literature on these topics continues, a historical perspective suggests that mutual engagement and understanding between these worlds has declined. The famous painting of Pinel unchaining the inmates of the Salpêtrière, painted in 1895, illustrates not a consequence of the French Revolution but the benefits of an empirical psychiatry based on observation that prioritises patient benefit without presuming prior theory.^2,3^ This active, empirical approach still characterises psychiatry.^4^ Nevertheless, psychiatrists are now represented as theorisers, more interested in investigation than benefiting our patients,^5^ and sometimes entirely indifferent to them.

As the humanities’ awareness and understanding of psychiatrists has diminished, so has our involvement with them. The conjunction of psychiatric, surrealist and philosophical thought between the 1920s and 1960s contributed to the development of both postmodernist thinking and antipsychiatry.^6^ Yet, even the memory of those connections now seems lost to us, and we are invited to consider them as if they are alien to our tradition and we had never responded.^7,8^

To encourage a rapprochement and interchange that takes into account the progress made since the middle of last century, we will seek to publish not only cultural thinking by psychiatrists but also work by practitioners of other methodologies. It is often forgotten that the visual arts, music, poetry and literature are also methods for exploring the world, especially our subjectivities. For psychiatry, the subjectivity of our patients is part of our core business, and we are no longer so restricted by the limits of paper and physical printing. We therefore do not necessarily require that a submission to the culture section is in the form of a conventional academic paper, provided that it addresses a cultural issue that has an impact on psychiatric practice and meets our quality standards.

### Regulatory culture

The arts and humanities are often seen as a counterpoise to excessive regulation. However, there is also regulatory culture, which operates to deliver the intentions of a regulatory regime when circumstances are indeterminate and discretion is essential.^9^ It can be thought of as the set of explicit or implicit attitudes and intentions expressed through norms, routine policy and everyday practice. It has become an explicit part of financial regulation, and firms are reviewed to ensure that their management structures deliver it.^10^ Within the National Health Service (NHS), regulatory culture is much more variably instituted^11^ and an audit model may not capture many of its necessary components.^12^ We therefore wish to publish articles relevant to improving the regulatory culture of psychiatric care, as the level of variation found suggests an ongoing and urgent need.

### The cultured practitioner

The mission of *BJPsych Bulletin* focuses on topics that affect what we do in our daily practice. The norms and values that our culture instils do precisely this. For example, social constructs such as masculinity and societal power gradients predict the balance between a response style of decisiveness versus accommodation.^13^ The concept of specifying moral principles in practical, situational terms is well-established in biomedical ethics.^14^ The same is needed for cultural influences, as professional practice cannot be detached from its cultural environment.^15^ Patient (service user) groups have begun developing this from an antipsychiatry perspective.^16^ We believe that practitioners need explicit accounts of how psychiatrists should express our discipline's best culture in daily practice. We will seek articles that relate the practice of psychiatrists to aspects of culture, applied to the benefit of their patients.

### Culture and society

Psychiatrists are taught their discipline as applied science. However, it is also one of society's institutions, tasked with performing an essential role. The Parthenon can be described entirely in engineering and aesthetic terms, but those perspectives do not explain how the Parthenon functioned. We also need to know that it was a Greek temple to Athena, the tutelary goddess of ancient Athens. To properly understand what our discipline delivers, and why, we need to be aware of how it is situated in our culture, how it maintains itself and the drivers that shape the services it delivers. Science is but one of these, and funding not only limits policy but also follows it. We are therefore interested in publishing articles that will let us map psychiatry's ‘cultural geography’, for example patterns of influence with other institutions, such as the law and politics, cultural drivers of research or service prioritisation, or the role of the relationship between psychiatrists and patients in shaping our clinical culture. We believe that having a clearer account of these will enable our profession to develop and deliver more effective services.

## Quality assurance

Cultural commentary from many perspectives is becoming increasingly widely distributed (e.g. medium.com; quillette.com) and, without expertise, quality can only be judged on its language and plausibility. Even ‘fact-checking’ may fail when, as often occurs in cultural scholarship, accuracy lies in the awareness of multiple interpretations, rather than allegiance to one. *BJPsych Bulletin* has two great strengths as a forum for cultural research and scholarship in psychiatry. Being open access, it has a potential reach similar to that of the online commentaries just mentioned. However, it also has a mature peer-review system. This combination gives it the potential to become the cultural ‘journal of record’ for our profession, as peer review will be applied to all the section's submissions, and articles and correspondence can be published from outside the profession. As we have seen above, at present, our cultural memory may be too short. Given the section's intended scope, psychiatrists will not be in a position to judge the quality of all potential submissions. Therefore, we plan to create a panel of reviewers covering the full range of methodologies, including the arts, to ensure that all articles will be evaluated by a respected peer in the field, as well as receiving a psychiatric review.

## Submitting articles

Articles should be submitted as ‘Cultural reflections’. Since the section is new, as the Culture Editor I will be pleased to discuss possible submissions at an early stage with potential authors. The purpose of such discussions is to ensure that the submission is in a form that meets the goals of the section and is suitable for forwarding for peer review.

## Our hope for the future

I have argued above that psychiatry needs to engage with culture to deliver best practice. I hope that the new section will support this by helping to establish cultural studies of all kinds as being of practical value to service delivery and demonstrating that the evidential quality of these studies is comparable with the best of scientific research.

## About the author

**David Foreman** is an affiliate senior lecturer in the Institute of Psychiatry, Psychology & Neuroscience, King's College London, UK. He is a member of the editorial board of *BJPsych Bulletin* and the editor of the new culture section.
